# Prognostic significance of platelet‑to‑albumin ratio in patients with nasopharyngeal carcinoma receiving concurrent chemoradiotherapy: a retrospective study of 858 cases

**DOI:** 10.1186/s12885-024-12499-w

**Published:** 2024-06-25

**Authors:** Xin Hua, Fei Xu, Wei Shi, Zhi-Qing Long, Xin Huang, Fang-Fang Duan, Si-Fen Wang, Chao Zhang, Meng-Di Wang, Wei-Qiong Ni, Wen Xia, Jia-Yi Chen, Yun-Sheng Gao

**Affiliations:** 1 Department of Radiation Oncology, Shanghai Jiao Tong University Medical School Affiliated Ruijin Hospital, Shanghai, Shanghai, China; 2 Department of Oncology, The Second Affiliated Hospital of Anhui Medical University, Hefei, China; 3 State Key Laboratory of Oncology in South China, Guangdong Provincial Clinical Research Center for Cancer, Guangdong Key Laboratory of Nasopharyngeal Carcinoma Diagnosis and Therapy, SunYat-sen University Cancer Center, Guangzhou, China

**Keywords:** PAR, Nomogram, Prognosis, Concurrent chemoradiotherapy, Nasopharyngeal carcinoma

## Abstract

**Background:**

Despite evidence supporting the high correlation of the novel platelet-to-albumin ratio (PAR) with survival in diverse malignancies, its prognostic relevance in nasopharyngeal carcinoma (NPC) remains underexplored. This study aimed to examine the link between PAR and overall survival (OS) in NPC and to establish a predictive model based on this biomarker.

**Methods:**

We retrospectively assembled a cohort consisting of 858 NPC patients who underwent concurrent chemoradiotherapy (CCRT). Utilizing the maximally selected log-rank method, we ascertained the optimal cut-off point for the PAR. Subsequently, univariate and multivariate Cox proportional hazards models were employed to discern factors significantly associated with OS and to construct a predictive nomogram. Further, we subjected the nomogram’s predictive accuracy to rigorous independent validation.

**Results:**

The discriminative optimal PAR threshold was determined to be 4.47, effectively stratifying NPC patients into two prognostically distinct subgroups (hazard ratio [HR] = 0.53; 95% confidence interval [CI]: 0.28–0.98, *P* = 0.042). A predictive nomogram was formulated using the results from multivariate analysis, which revealed age greater than 45 years, T stage, N stage, and PAR score as independent predictors of OS. The nomogram demonstrated a commendable predictive capability for OS, with a C-index of 0.69 (95% CI: 0.64–0.75), surpassing the performance of the conventional staging system, which had a C-index of 0.56 (95% CI: 0.65–0.74).

**Conclusions:**

In the context of NPC patients undergoing CCRT, the novel nutritional-inflammatory biomarker PAR emerges as a promising, cost-efficient, easily accessible, non-invasive, and potentially valuable predictor of prognosis. The predictive efficacy of the nomogram incorporating the PAR score exceeded that of the conventional staging approach, thereby indicating its potential as an enhanced prognostic tool in this clinical setting.

**Supplementary Information:**

The online version contains supplementary material available at 10.1186/s12885-024-12499-w.

## Introduction

Nasopharyngeal carcinoma (NPC) represents a relatively rare malignancy with a disproportionate incidence in East and Southeast Asia, accounting for over 130,000 annual diagnoses globally, the majority of which (≥ 70%) present with locoregionally advanced disease [1, 2]. For locally advanced NPC, concurrent chemoradiotherapy (CCRT) is the standard therapeutic strategy [3, 4]. Presently, the tumor-node-metastasis (TNM) staging system serves as the cornerstone for predicting outcomes and directing treatment decisions [5, 6]. However, considerable interpatient variability in survival exists even among individuals with identical TNM stages, as evidenced by disease progression in up to 30% of patients despite similar treatment modalities [7, 8]. This underscores the inadequacy of TNM staging in fully capturing the biological diversity of NPC.

In recent times, the quest for robust biomarkers capable of accurate prognostic prediction and individualized risk stratification in NPC has intensified. Nutrition and inflammation, pivotal in cancer initiation and progression [9, 10], have driven investigations into composite biomarkers that integrate both aspects. Among these are the lymphocyte-C-reactive protein ratio (LCR), Controlling Nutritional Status (CONUT), prognostic nutritional index (PNI), Glasgow prognostic score (GPS), systemic immune-inflammation index (SII), monocyte-lymphocyte ratio (MLR), platelet-lymphocyte ratio (PLR), and neutrophil-lymphocyte ratio (NLR), which have gained traction in numerous cancers, including NPC [11–18]. Despite this, the relationship between pretreatment PAR and survival in NPC patients, particularly those undergoing CCRT, remains poorly understood.

This study addresses the paucity of evidence on PAR’s prognostic significance in NPC patients receiving CCRT. We sought to explore the correlation between pretreatment PAR levels and clinical outcomes in these patients.

## Materials and methods

### Patients

Patients who underwent platinum-based CCRT at Sun Yat-sen University Cancer Center between January 2010 and December 2014 were included in this retrospective analysis. The criteria for inclusion were:

(I) confirmation of treatment-naive, non-metastatic NPC using radiographic and histological assessments;
(II) patients who had Epstein-Barr virus (EBV) DNA tests and peripheral blood and serum laboratory pretreatment data;
(III) patients who received weekly/triweekly concurrent chemotherapy with platinum-based drugs and radiotherapy treatment with radical intensity modulation, patients treated with induction chemotherapy were excluded.
(IV) patients who have no malignancy history and have never suffered from acute or chronic inflammatory diseases, including concomitant diseases that may affect platelet count or albumin level, such as autoimmune disease, history of blood transfusion, liver cirrhosis, severe inflammation or infection in the past month. Patients having received anticoagulant therapy or infused albumin before blood collection also excluded.

The 8th AJCC TNM system was used to update the staging of all patients. Sun Yat-sen University Cancer Center’s Ethics Committee approved the study and waived written informed consent since the research was retrospective. All procedures were carried out in strict compliance with all applicable rules and regulations (adhering to the principles stipulated in the 1964 Declaration of Helsinki and any subsequent amendments thereto, or to comparable ethical standards).

### Acquisition of data and follow-up

Within seven days following the diagnosis, the primary laboratory data were obtained and patients’ medical records were screened for clinical and pathological data. Plasma EBV-DNA levels (copies/ml) were measured via real-time quantitative polymerase chain reaction (RT-qPCR). Then, the body mass index (BMI) was calculated as weight (kg) divided by the square of height (m^2^). Subsequently, the patients were determined to different weight groups: BMI ≥ 24, BMI 24–28 or BMI >28. The treatment and follow-up procedures were carried out in adherence to the guidelines that were previously defined [19, 20]. Overall survival (OS) was defined as the time from the date of diagnosis to the date of death or last follow-up.

### Statistical analysis

Owing to the lack of data supporting the development of prognostic models, prior sample size calculations were absent. Out of the 858 individuals that were recruited, 68 events were recorded during the current investigation. This is an excess rate of 10 events per variable in the multivariate models, which demonstrates that there was enough power for evaluation [21]. Log-rank method was used to effectively discriminate by maximizing the difference between patient survival curves identify the cut-off value for PAR [22]. The “maxstat” package was utilized to calculate the optimal cutoff value based on the maximum specified rank statistics with the endpoint being the survival status [23]. The survival curves were created via the Kaplan-Meier technique and then contrasted by log-rank tests. The proportional hazards hypothesis was examined with Schoenfeld residuals. Univariate and multivariate analyses were conducted utilizing the Cox proportional hazards model and only variables that had a *P* value < 0.10 in the univariate analysis were subjected to the multivariate analysis. Next, nomograms were developed utilizing data from the multivariate analysis. The nomogram’s performance was assessed utilizing the calibration curve, C-index, and area under the curve (AUC) of the tROC analysis. Statistical significance was determined by a two-tailed *P* < 0.05. We employed R 4.2.1 to perform our statistical analyses.

## Results

### Characteristics of the patients

Participants in this research included 858 patients with NPC who had platinum-based CCRT at the Sun Yat-sen University Cancer Center from January 2010 to December 2014. Table 1 provides an overview of the clinicopathological features at baseline. Notably, 638 (74.4%) of the total patients were male, while 220 (25.6%) were female, with 64 years being the median age (range: 18–84 years). There were 436 (50.8%) patients > 45 years old and 422 (49.2%) patients under the age of 45. A pathological diagnosis of histological type WHO III was confirmed for most included patients and 278 (32.4%) had an EBV-DNA value ≥ 4000 copy/ml. Then, patients were grouped into high-PAR (scored>4.47, *n* = 607) and low-LCR (scored ≤ 4.47, *n* = 251) categories, according to the maximally selected rank statistics-established optimal PAR cutoff value of 4.47 (Figure S1).

**Table 1:** Patient demographics and clinical variables of the study participants

Characteristic	All (*n* = 858)	%
Age		
≥ 45 years	436	50.8
<45 years	422	49.2
Gender		
Male	638	74.4
Female	220	25.6
Histological type		
WHO I/II	13	1.5
WHO III	845	98.5
HGB		
<113 g/L	27	3.2
113–151 g/L	546	63.6
≥ 151 g/L	285	33.2
LDH		
≥ 245 U/L	807	94.1
<245 U/L	51	5.9
ALB		
≥ 40 g/L	786	91.6
<40 g/L	72	8.4
T stage		
T1	41	4.8
T2	165	19.2
T3	523	61.0
T4	129	15.0
N stage		
N0	81	9.4
N1	463	54.0
N2	270	31.5
N3	44	5.1
BMI		
≤ 24 kg/m^2^	517	60.3
24–28 kg/m^2^	293	34.1
≥ 28 kg/m^2^	48	5.6
EBV-DNA		
<4000 copy/ml	580	67.6
≥ 4000 copy/ml	278	32.4
Platelet counts		
≤ 300 × 10^9^/L	742	86.5
>300 × 10^9^/L	116	13.5
PAR		
>4.47	607	70.7
≤ 4.47	251	29.3

Abbreviations: WHO = World Health Organization; HGB = hemoglobin; LDH = serum lactate dehydrogenase levels; BMI = body mass index; EBV-DNA = Epstein-Barr virus DNA; PAR = Platelet‑to‑Albumin ratio

### Significance of the PAR score in predicting OS in NPC

**Fig. 1:**
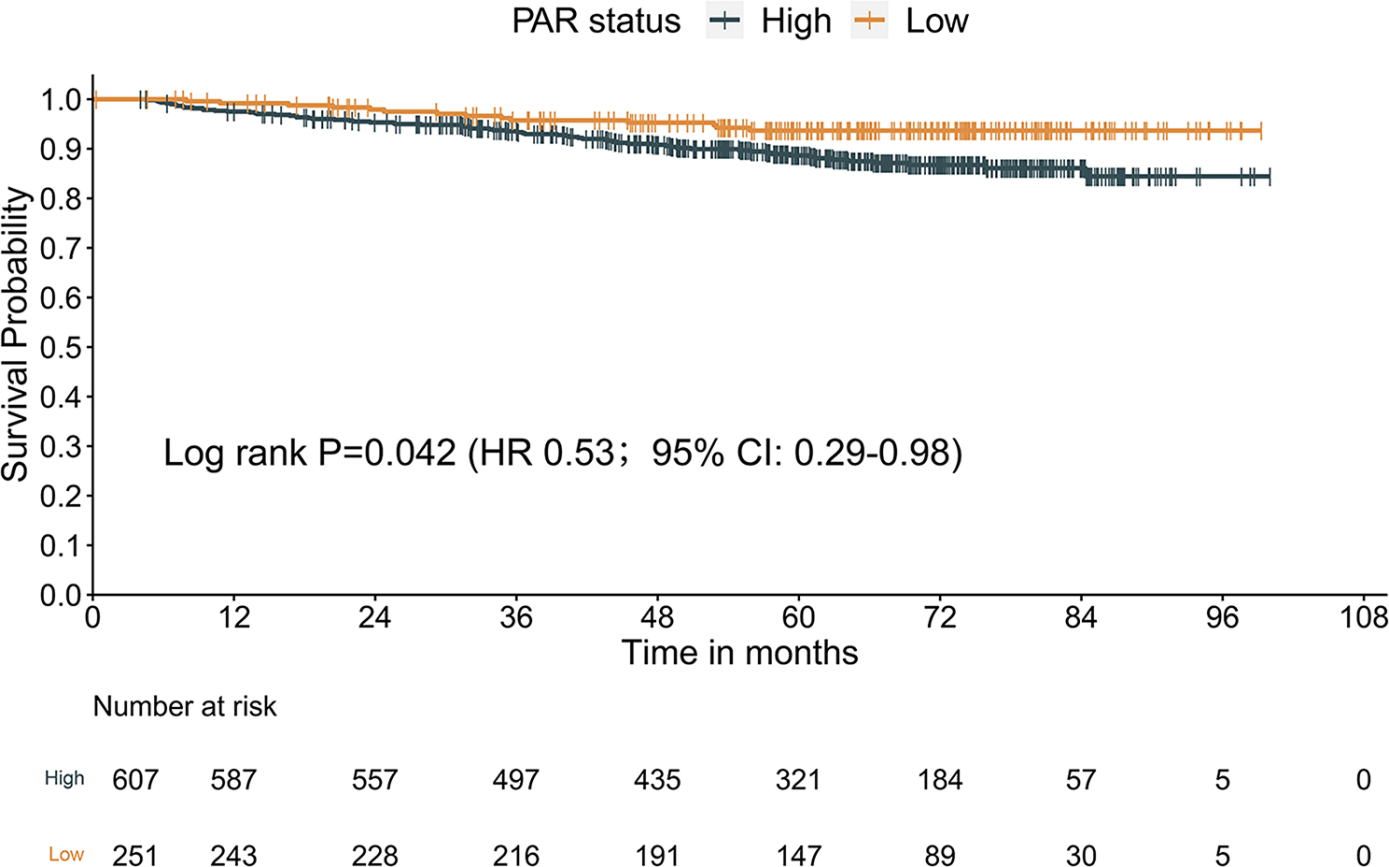
Survival curves obtained with Kaplan-Meier analysis between different PAR Groups (the HRs reported were unadjusted). Abbreviations: PAR = Platelet‑to‑Albumin ratio; HR = hazard ratios; CI = confidence interval

The median OS was 62.5 months (IQR: 46.6–74.8 months). A total of 83 events were documented within the study period. The respective OS rates for 1-, 3-, and 5-year were 98.0%, 95.9%, and 93.8%. Survival was significantly increased for patients with low PAR relative to those with high PAR, as demonstrated by Kaplan-Meier curves (Fig. 1, HR = 0.53; 95% CI: 0.29–0.98, *P* = 0.042).

### Univariate and multivariate Cox regression analyses of OS in NPC

The multivariate Cox model included factors like age, EBV-DNA status, PAR score, N stage, T stage, and histology that satisfied the predetermined significance criterion (*P* < 0.10) in the univariate model. A diagnostic test for multicollinearity was performed by computing the variance inflation factors (VIFs) of the aforementioned variables (all VIFs were less than 10). The test results confirmed the absence of severe multicollinearity. The multivariate model satisfies the proportional hazards assumption, as indicated by the proportional hazard’s diagnostic charts (Figure S2). Notably, T stage, N stage, and PAR score were shown to be independently linked to OS for patients with NPC undergoing CCRT (Table 2).

**Table 2:** Univariate and multivariate Cox regression analyses of overall survival

Characteristic	Univariate analysis		Multivariate analysis		
	Hazard ratio(95%CI)	P	Hazard ratio(95%CI)	*P*	
Age					
≥ 45 years	1		1		
<45 years	1.598(0.985–2.594)	0.058	0.652(0.420–1.012)	0.057	
Gender					
Male	1				
Female	0.751(0.417–1.353)	0.341			
Histological type					
WHO I/II	1		1		
WHO III	0.34(0.107–1.084)	0.068	0.424(0.131–1.380)	0.154	
HGB					
<113 g/L	1				
113–151 g/L	1.889(0.259–13.761)	0.530			
≥ 151 g/L	2.704(0.368–19.857)	0.328			
LDH					
≥ 245 U/L	1				
<245 U/L	0.714(0.287–1.778)	0.470			
T stage					
T1	1		1		
T2	2.587(0.331–20.232)	0.365	2.955(0.383–22.797)	0.299	
T3	3.362(0.462–24.472)	0.231	3.842(0.529–27.916)	0.184	
T4	5.605(0.743–42.276)	0.095	8.014(1.072–59.905)	0.043	
N stage					
N0	1		1		
N1	1.709(0.517–5.648)	0.379	1.598(0.563–4.534)	0.378	
N2	3.538(1.081–11.577)	0.037	3.028(1.059–8.660)	0.039	
N3	4.509(1.123–18.11)	0.034	4.136(1.230-13.909)	0.022	
BMI					
≤ 24 kg/m^2^	1				
24–28 kg/m^2^	0.803(0.473–1.363)	0.417			
≥ 28 kg/m^2^	0.963(0.346–2.685)	0.943			
EBV-DNA					
<4000 copy/mL	1		1		
≥ 4000 copy/mL	1.694(1.047–2.742)	0.032	1.248(0.789–1.975)	0.343	
PAR					
>4.47	1		1		
≤ 4.47	0.534(0.291–0.979)	0.042	0.502(0.282–0.896)	0.020	

Hazard ratios estimated by Cox proportional hazards regression. All statistical tests were two-sided. Abbreviations: WHO = World Health Organization; HGB = hemoglobin; LDH = serum lactate dehydrogenase levels; BMI = body mass index; EBV-DNA = Epstein-Barr virus DNA; PAR = Platelet‑to‑Albumin ratio

### Creation of a novel PAR-based prognostic model

**Fig. 2:**
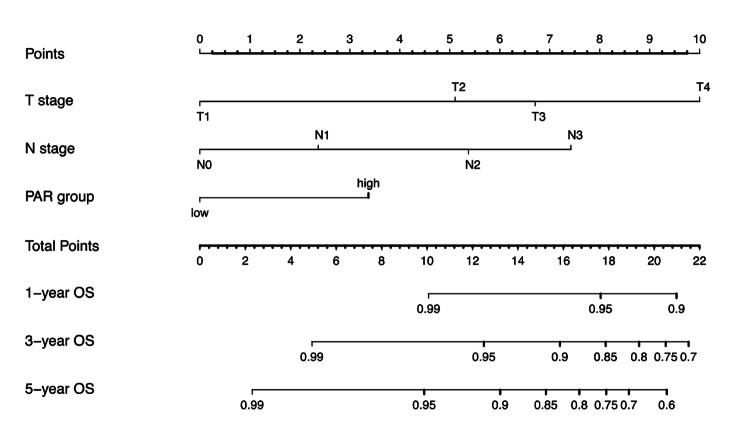
Nomogram of the current prognostic model for individualized survival predictions. Abbreviations: OS = overall survival; PAR = platelettoalbumin ratio

Using the aforementioned four independent variables derived from the multivariate model, a novel nomogram prognostic model was established to predict patient survival at 1, 3, and 5 years (Fig. 2). Before the introduction of CCRT, the scores of each of the 4 prognostic factor subtypes were added to derive the patient’s total score, and the probability of survival at 1, 3, and 5 years was predicted by placing the total score on the survival rate scale. For instance, one of the patients presented with T2 stage, N3 stage, and PAR score >4.47, the total point was: 5.1 + 7.5 + 3.5 = 16.1, and the respective 1-, 3-, and 5-year OS rates were 96%, 89%, and 81%.

### Evaluation of the prediction efficacy of the prognostic model

**Fig. 3:**
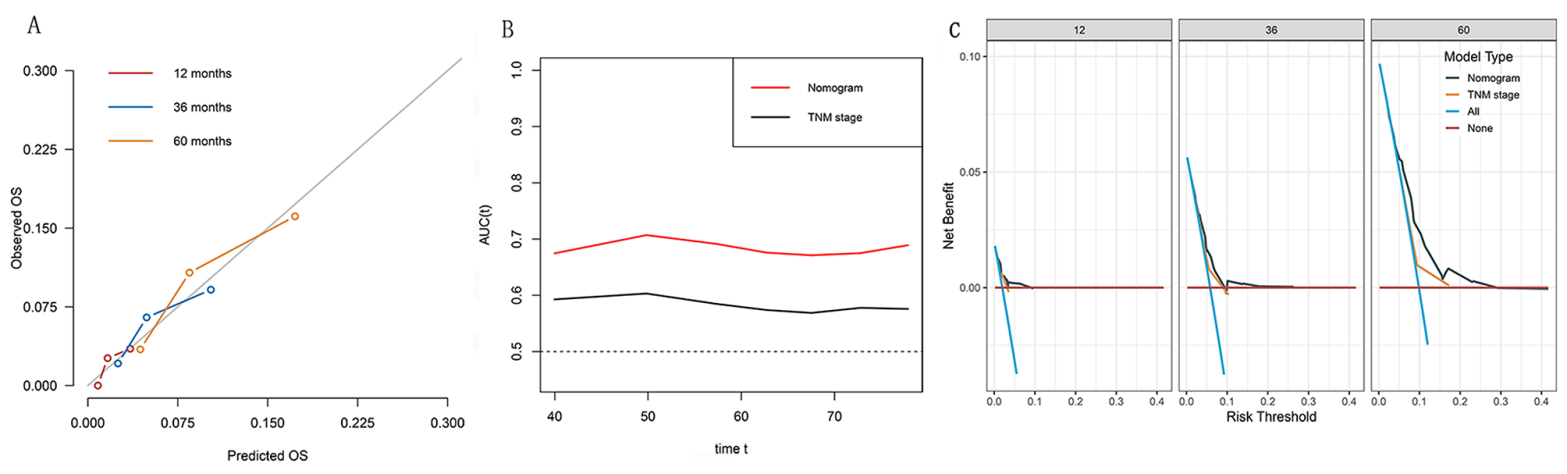
Assessment of predictive performance of the prognostic model. **(A)** Calibration plot of the nomogram model at 1, 3, and 5 years. **(B)** Time-independent ROC curves compared the predictive accuracy of the current model and the traditional TNM stage. **(C)** DCA curves compared the net benefit rate of the current model and the traditional TNM stage. Abbreviations: OS = overall survival; AUC = area under the curve; TNM = tumor node metastasis

The OS nomogram demonstrated satisfactory discrimination. The nomogram yielded C-indexes of OS [C-index = 0.69 (95% CI: 0.64–0.75)] and was superior to the conventional TNM staging system [C-index = 0.56 (95% CI: 0.65–0.74)]. Strong agreement was shown between predicted and actual OS in the calibration plots for the 1-, 3-, and 5-year OS (the actual survival is denoted along the ordinate, whilst the nomogram predicted survival is shown along the abscissa) (Fig. 3). Based on the OS after 1-, 3-, and 5-year follow-ups, the AUC values of the nomogram (0.72, 0.66, and 0.67) were higher than those of the TNM staging system (0.62, 0.58, and 0.59) (Fig. 3).

## Discussion

We examined whether the pretreatment PAR could predict the OS independently in NPC patients who had undergone CCRT in this study. Our analysis highlighted a significant improvement in the OS of patients with a low pretreatment PAR (< 4.47 ) relative to those with a high pretreatment PAR (≥ 4.47). The PAR was confirmed to be an independent marker of OS via multivariate analysis. The novel nutrition-inflammation marker of PAR and a PAR-based prognostic model were developed since the traditional anatomical TNM staging method is inadequate to predict NPC patients’ prognoses.

The prognosis differs among similarly-TNM-staged NPC, revealing that the TNM staging system presents insufficient as a comprehensive staging system to differentiate tumor heterogeneity. Introducing additional relevant biomarkers such as inflammatory-nutritional factors into the TNM staging system could enhance the predictive accuracy of clinical outcomes. An elevated platelet count is often associated with a higher risk of various solid cancers and is linked to poorer outcomes in clinical trials [24, 25]. Chen et al. demonstrated that thrombocytosis was correlated with a dismal OS and was more common in those whose illness had progressed to advanced stages in 2626 patients with NPC [26]. Hypoalbuminaemia in patients treated with malignancy also indicates poor clinical outcomes. Previous research has shown that pretreatment hypoalbuminemia is linked to a decreased survival rate in various cancer types [27]. A meta-analysis published in 2020 including 10 studies of 7339 NPC patients also found that a lower serum level pretreatment of ALB concentration implied a worse prognosis of OS(HR = 1.32, 95% CI 1.17–1.48) [28]. Hypoalbuminaemia could also contribute to a high PAR. Based on the above previous researches, an elevation of PAR contributing by increased platelet count or/ and decreased serum albumin level, might be a useful indicator of the prognosis of malignant tumors. In 2019, Shirai, Y. et al. identified PAR as a novel nutrition-inflammation-based prognostic score that predicted DFS and OS in patients after pancreatic resection [29]. In this retrospective study, we uncovered a distinct correlation between pretreatment PAR score and OS among 858 NPC patients receiving CCRT. The results revealed that a pretreatment PAR score ≥ 4.47 was statistically linked with significantly reduced OS (HR: 0.53, 95% CI: 0.29–0.98, *P*-value = 0.042), thereby substantiating its potential as a predictive biomarker for clinical outcomes in this patient population. In addition, we established a PAR-based predictive nomogram model, integrated with TNM staging, that offers a greater prognostic predictive ability compared to TNM staging alone.

Since thrombocytosis platelet plays considerable roles in inflammation reflection and immune response [30]. As of yet, the etiology of tumor-associated thrombocytosis is undetermined. One hypothesis is that platelet activation may be induced by cytokines produced by cancer cells, such as transforming growth factor-β1 (TGF-β1), vascular endothelial growth factor (VEGF), and platelet-derived growth factor (PDGF). These platelet-derived cytokines are pivotal to tissue proliferation, angiogenesis, and metastasis, all of which are critically involved in the development of tumors [31]. Besides, platelets may enhance tumor cell arrest at the endothelium and protect them from host immune system assault [32–34]. Hypoalbuminemia typically indicates malnutrition status and implies weakened immune defense systems. A subdued immune system could lead to activated systemic inflammatory responses that promote cancer progression, releasing inflammatory factors such as tumor necrosis factor, interleukin-1, and C-reactive protein, which may be associated with the inhibition of the synthesis of albumin in hepatocytes [35, 36]. On the other hand, albumin has been proven to have antitumor [37, 38] and antioxidant effects [39]. Hypoalbuminaemia is considered to set the scene for cancer, and cancer further lowers albumin [40]. These results of mechanism researches may offer the explanation as to why a high level of PAR can result in unfavorable outcomes.

EBV DNA loads(with a cutoff value of 4000 copies/mL) is considered to predict survival outcomes in NPC patients. Lan et al. illustrates EBV DNA’s superiority over TNM staging in predicting progression-free survival (PFS), distant metastasis-free survival (DMFS), and locoregional relapse-free survival (LRRFS), whereas the predictive value of the combination of pre-DNA and mid-DNA on OS is lower than that of the TNM stage [41]. In our research, while EBV DNA demonstrated significance in univariate analysis, it lost significance in the multivariate model, which could partly be attributed to complex biological interactions between EBV DNA and other clinical-pathological features, like collinearity with T and N, although the Variance Inflation Factors (VIFs) for T and N at 6.8 and 9.5, respectively, suggest collinearity below the commonly accepted threshold of statistical concern (VIF < 10). Also, our previous study [42] on sarcopenia and NPC prognosis also found that EBV-DNA levels were significant in univariate but not in multivariate analysis for OS. To better elucidate EBV DNA’s role in NPC prognosis, further additional analyses through increasing sample size to enhance statistical power or conducting multi-center studies to validate EBV DNA’s prognostic value need to plan to refine our present research.

Additionally, the age, N stage, and T stage were other independent prognostic indicators correlated with OS for NPC patients treated with CCRT. These results are consistent with previous research results [18, 43]. Therefore, we combined these independent factors with the simplified, affordable, and non-invasive serum markers to develop a nomogram to predict OS in NPC patients, which might offer a potential guide for individual clinical care. For example, a more intensive treatment approach might be required in patients with high scores using the nomogram model system, with anticoagulants or human serum albumin as support treatment plan.

### Limitations

The limitations of this investigation are: Firstly, the retrospective design inherently introduced an element of selection bias, which could potentially confound the results. Secondly, the development of the prognostic model relied solely on a patient cohort derived from a single institution, may underscoring the necessity for rigorous external validation using high-quality data procured from multiple centers. Furthermore, the variability of PAR status across time and its susceptibility to influence from diverse clinical contexts represent additional sources of complexity that were not fully accounted for in this analysis. Moreover, to strengthen the conclusiveness of our findings, future endeavors include plans to accumulate supplementary data for dynamic assessment and to embark upon a prospective multicenter research endeavor aimed at corroborating these preliminary observations. This strategic approach seeks to address the current limitations and enhance the generalizability and robustness of our proposed prognostic model.

## Conclusions

In the context of NPC patients undergoing CCRT, the novel nutritional-inflammatory biomarker PAR emerges as a promising, cost-efficient, easily accessible, non-invasive, and potentially valuable predictor of prognosis. The predictive efficacy of the nomogram incorporating the PAR score exceeded that of the conventional staging approach, thereby indicating its potential as an enhanced prognostic tool in this clinical setting. A predictive nomogram model was thus established, providing a simple way to group patients into different treatment reaction of CCRT.

### Electronic supplementary material

Below is the link to the electronic supplementary material.

Supplementary Material 1

## Data Availability

No datasets were generated or analysed during the current study.

## Abbreviations

PAR: platelet-to-albumin ratio
NPC: nasopharyngeal carcinoma
OS: overall survival
CCRT: concurrent chemoradiotherapy
LCR: lymphocyte-C-reactive protein ratio
CONUT: Controlling Nutritional Status
PNI: prognostic nutritional index
GPS: Glasgow prognostic score
SII: systemic immune-inflammation index
MLR: monocyte-lymphocyte ratio
PLR: platelet-lymphocyte ratio
NLR: neutrophil-lymphocyte ratio
EBV: Epstein-Barr virus
RT-qPCR: real-time quantitative polymerase chain reaction
BMI: body mass index
AUC: area under the curve
IQR: interquartile range
VIFs: variance inflation factors
TGF-β1: transforming growth factor-β1
VEGF: vascular endothelial growth factor
PDGF: platelet-derived growth factor
