# Supplementary material for: Prognostic significance of platelet‑to‑albumin ratio in patients with nasopharyngeal carcinoma receiving concurrent chemoradiotherapy: a retrospective study of 858 cases
Source: BMC Cancer. 2024 Jun 25;24:762. doi: 10.1186/s12885-024-12499-w (PMC11197365; doi:10.1186/s12885-024-12499-w)
Supplement: Supplementary file 1 — Supplementary Material 1 [file 12885_2024_12499_MOESM1_ESM.docx]

**Figure S1. Derivation of the cutoff value of the PAR score according to maximally selected log-rank statistics.**

**Figure S2. Proportional hazards diagnostic plots of multivariable Cox modeling.**
